# Whether, when, how, and how much? General public’s and cancer patients’ views about the disclosure of genomic secondary findings

**DOI:** 10.1186/s12920-021-01016-8

**Published:** 2021-06-26

**Authors:** Jude Emmanuel Cléophat, Michel Dorval, Zaki El Haffaf, Jocelyne Chiquette, Stephanie Collins, Benjamin Malo, Vincent Fradet, Yann Joly, Hermann Nabi

**Affiliations:** 1 Faculty of Pharmacy, Laval University, Quebec City, QC Canada; 2 Oncology Division, Research Center of the CHU de Québec-Laval University, Hôpital du Saint-Sacrement, 1050, chemin Sainte-Foy, Québec, QC G1S 4L8 Canada; 3 Laval University Cancer Research Center, Quebec City, QC Canada; 4 Research Center of the Chaudière-Appalaches Integrated Center for Health and Social Services, Lévis, QC Canada; 5 Division of Genetics, Department of Medicine, Hospital Center of the University of Montreal, Montreal, QC Canada; 6 Oncology Division, Research Center of the Hospital Center of the University of Montreal, Montreal, QC Canada; 7 Department of Family Medicine and Emergency Medicine, Faculty of Medicine, Laval University, Quebec City, QC Canada; 8 Faculty of Pharmacy, University of Montreal, Montreal, QC Canada; 9 Infectious and Immune Diseases Division, Research center of the Quebec City University Hospital, Quebec City, QC Canada; 10 Department of Surgery, Faculty of Medicine, Laval University, Quebec City, QC Canada; 11 Center of Genomics and Policy, McGill University, Montreal, QC Canada; 12 Department of Social and Preventive Medicine, Faculty of Medicine, Laval University, Quebec City, QC Canada

**Keywords:** Focus groups, Genomics, Secondary findings, Genetic testing, Genome sequencing, Confidentiality, Canada, Disclosure, Qualitative research, Neoplasm

## Abstract

**Background:**

Data on the modalities of disclosing genomic secondary findings (SFs) remain scarce. We explore cancer patients’ and the general public’s perspectives about disclosing genomic SFs and the modalities of such disclosure.

**Methods:**

Sixty-one cancer patients (n = 29) and members of the public (n = 32) participated in eight focus groups in Montreal and Quebec City, Canada. They were asked to provide their perspectives of five fictitious vignettes related to medically actionable and non-actionable SFs. Two researchers used a codification framework to conduct a thematic content analysis of the group discussion transcripts.

**Results:**

Cancer patients and members of the public were open to receive genomic SFs, considering their potential clinical and personal utility. They believed that the right to know or not and share or not such findings should remain the patient’s decision. They thought that the disclosure of SFs should be made mainly in person by the prescribing clinician. Maintaining confidentiality when so requested and preventing genetic discrimination were considered essential.

**Conclusion:**

Participants in this study welcomed the prospect of disclosing genomic SFs, as long as the right to choose to know or not to know is preserved. They called for the development of policies and practice guidelines that aim to protect genetic information confidentiality as well as the autonomy, physical and psychosocial wellbeing of patients and families*.*

**Supplementary Information:**

The online version contains supplementary material available at 10.1186/s12920-021-01016-8.

## Background

The advent of high-throughput platforms has made it possible to sequence billions of DNA base-pairs faster and at lower costs than conventional sequencing [1]. New sequencing technologies offer several advantages and may be used for several purposes, particularly in personalizing oncology treatments [1–4]. Despite the potential benefits of sequencing technology, its use in clinical practice may raise some issues [5]. Indeed, secondary findings (SFs) from exome/genome sequencing may predict drug response or toxicity and reveal a predisposition to serious medical conditions in tested individuals [6, 7]. In some cases, such findings may help in disease prevention and treatment adjustment [8]. In others, preventive or therapeutic options may not be available. Being aware of genetic susceptibility to a serious illness, for which there is no treatment, can psychologically affect patients and their families at least in the short term [9].

Genetics professional societies are divided on the extent to which SFs should be investigated and communicated to patients. In its 2016 policy statement, the American College of Medical Genetics and Genomics (ACMG) advocated that, when genome sequencing is ordered, 59 variants associated with 27 treatable diseases or disease groups could be searched and disclosed, regardless of patient’s age and reason for consultation [10]. The ACMG also offers patients the option to refuse the analysis or the reception of SFs related to the 59 variants above [10, 11]. The European Society of Human Genetics (ESHG) and the Canadian College of Medical Geneticists (CCMG) favor the targeted search for actionable and highly penetrant variants in sets of genes directly related to the primary clinical issue to avoid discovering unexpected or uninterpretable variants [4, 12]. This lack of consensus in legislations and recommendations shows the complexity of conveying genomic SFs to patients.

Several quantitative and qualitative studies have shown that patients, healthcare professionals, and the lay public tend to have positive attitudes about disclosing genomic SFs both in research and clinical settings [13, 14]. However, the type of results that should be generated and made available to patients remains less clear [13]. Indeed, while professional genetics societies recommend exclusively disclosing SFs, for which prevention and treatment are available, tested individuals might be willing to receive SFs related to untreatable or unpreventable medical conditions [14]. This would widen the spectrum of disclosable SFs and complexify the modalities of such disclosure. Moreover, the moment, manner, and type of provider who discloses SFs might be important to some patients, particularly those already dealing with a serious disease [15–19]. Nonetheless, stakeholders’ preferences regarding such questions have been little explored. Finally, the type of support that prospective tested individuals need concerning SFs disclosure has also received little attention so far. The objective of the present study was to explore the perspectives of cancer patients and healthy members of the public about disclosing genomic SFs and, the modalities of such disclosure. Our specific research questions were:

(1) What are the opinions and attitudes of cancer patients and healthy members of the public about the disclosure of genomic SFs?

(2) What are their preferences regarding the conditions and methods of disclosure of these SFs?

(3) What are their perceptions of the potential psychosocial implications of knowing these SFs, and their support needs in such matters.

## Materials and methods

### Participants

A focus group study was conducted targeting cancer patients and members of the public. The choice to include cancer patients is justified by the fact that oncology is a fertile ground for the development and applications of genetics and genomics, since the development of cancers is often linked to genomic changes [20]. As a result, some cancer patients might have better genomic literacy than individuals from general population. It is therefore important to have their perspectives alongside those from the general population for the sake of diversity in points of view. Cancer patients, who may have undergone prior genetic testing, were identified in three sites: the *Deschênes-Fabia* Breast Diseases Center and the Department of Urologic Oncology of the Quebec City University Hospital, and the Department of Genetic Medicine of the Montreal University Hospital Center. Potential participants were approached during a follow-up visit or called by a research assistant or nurse who explained the study, confirmed their eligibility and obtained their written consent. Cancer-affected participants’ contact information was forwarded to SOM (https://www.som.ca/en), the private research firm in charge of organizing and conducting the focus groups. SOM is a Quebec-based research firm specializing in qualitative and quantitative research. This firm was selected following a rigorous tender process as we have successfully done in previous studies [21, 22]. Participants from the public were recruited through the research firm panel made up of people randomly recruited by telephone, never diagnosed with cancer. All prospective participants received a confirmation letter and a phone call the day before the focus group to confirm their presence. Participants from the public signed the consent form at the beginning of the focus groups.

### Data collection

Considering the controversial nature of the debate surrounding the disclosure of genomic SFs, focus groups appear to be the most suitable method for exploring perspectives, revealing preferences, and understanding around social issues related to this question [23, 24]. Eight focus group discussion sessions were conducted from April to July 2018. Four sessions were held with cancer patients (two with men and two with women) and four with members of the public (two with men and two with women). These group discussions were held in Montreal (n = 4) and Quebec City (n = 4) in a specially equipped room at the research firm facility. An experienced female moderator conducted the discussions using a semi-structured discussion guide developed by the research team (Supplementary Document 2. Additional file 1: this document presents the discussion guide.). This guide includes a case example of a patient diagnosed with colon cancer presenting drug resistance and for whom genome sequencing was requested to investigate the origin of such resistance. Five hypothetical SFs from the patient’s genome sequencing were presented in five clinical vignettes. These vignettes revealed the patient’s predisposition to four medically actionable and non-actionable diseases, namely hypercholesterolemia, Alzheimer's disease, Wilson’s disease, cystic fibrosis, and a variant of unknown significance (VUS). The discussion guide contains questions that allow documenting participants’ opinions, attitudes, preferences, and psychological support needs regarding genomic SFs disclosure. They were also asked about policies and practice guidelines regarding SFs. At the beginning of each group, the moderator informed participants that she was not a member of the research team and that research team members would be observing discussions behind a one-way mirror. She reminded participants of the study objectives and provided them with basic information about genomics and SFs before presenting the case example and vignettes. The moderator asked participants to read the vignettes and pretend they were a cancer patient receiving the SFs disclosed in the vignettes. When needed, the moderator asked further open-ended questions. Each group discussion lasted about 2 h. They were audio and video recorded and transcribed verbatim. Each participant received $75 (CAD) compensation.

### Data analysis

A thematic content analysis of the transcripts was conducted. One member of the research team (SC) developed an initial codification framework based on the scientific literature, themes in the discussion guide, and study objectives. Following a deductive approach, two research team members (SC, BM) independently coded discussion transcripts and compared findings. Differences were resolved by consensus. Following an inductive approach [25], subthemes that emerged throughout the analysis were added to the initial codification framework. The codification process was assisted by QDA Miner (Provalis Research, Montreal, Canada), and coded excerpts were placed under their corresponding subtheme and theme. Excerpt findings were abstracted using the method developed by Miles and Huberman [26].

### Ethical considerations

The Ethics Review Boards of the *CHU de Québec* (Quebec City University Hospital) and the *CHU de Montréal* (Montreal University Hospital) approved this study. Participants provided informed consent before the focus groups. Their first names only were used to maintain confidentiality. Reported quotes were anonymized.

## Results

Table 1 presents the characteristics of the sample. Sixty-one cancer patients (n = 29) and members of the public (n = 32) participated in the focus groups. They were mostly female (56%). Fifty-one percent were aged over 50. A similar proportion had a college education. Participants worked in diverse fields.

**Table 1:** Participants’ characteristics

	Group 1	Group 2	Group 3	Group 4	Group 5	Group 6	Group 7	Group 8	Total N = 61
Participants									
Public	10	7	7	8					32
Patients					9	7	8	5	29
Location									
Québec City	10	7			9	7			33
Montréal			7	8			8	5	28
Gender									
Female	10		7		9		8		34
Male		7		8		7		5	27
Age groups									
25 or less	0	2	2	1	0	0	0	0	5
26 to 35	4	0	2	2	0	0	0	0	8
36 to 50	1	3	1	3	5	0	4	0	17
50 or more	5	2	2	2	4	7	4	5	31
Education									
High school	7	4	4	5	3	3	3	1	30
College	3	3	3	3	6	4	5	4	31

### The right to choose to know or not to know

Quotes related to this subsection are presented in Table 2. Participants generally agreed that patients should be given the choice between knowing and not genomic SFs. The ability to choose seemed even more important to participants when genomic SFs concerned a serious medical condition (Quote 1). They thought that the option to receive this information, or not, should be offered before prescribing the test, and separately for actionable, non-actionable findings, and VUS (Quote 2). Some participants stated that healthcare professionals should reveal SFs to their patients, particularly when these findings have implications for children (Quote 3). One participant revealed that she initially thought it was illegal not to share this type of information with patients (Quote 4).

**Table 2:** Quotes related to participants’ perceptions of the patients’ right to choose to know or not to know about genomic sequencing SFs

Selected quotes
Quote 1: “*It is more a question about the other person’s willingness to know; it comes from the patient’s point of view because it depends on his point of view. They are not going to force him (the patient) to tell him directly. If he doesn’t feel like knowing, it’s his body, and it’s him who decides if he wants to know or not what is going on or not, whether he wants to worry or not. That’s the person’s choice*.” Male from the general Quebec City population
Quote 2: “*No, I do think that the person should be able to choose beforehand. If it’s a diagnosis on which she can act, maybe she will be more inclined to want to know it. If it’s a diagnosis on which she’s powerless, I don’t see why I would impose it on her. If she decides that she doesn’t want to know, that’s her choice. But, I would respect her choice.*” Montreal female cancer survivor Quote 3: “*It’s good to have the choice. Also, if we have kids, to know…for the kids, the risks that they may incur in the future. It is not only about the patient’s responsibility (towards herself); there is also the responsibility towards her relatives*.” Montreal female cancer survivor
Quote 4: “*I was really surprised because to me it’s really the right to information so I even thought that from a legal point of view, it was like illegal not to inform me of the results… but I personally would like to know everything*” Woman from the Quebec City general population

### Motivations to know or not to know

Quotes related to participants’ motivations whether to receive genomic SFs or not are provided in Table 3. A detailed report of such motivations and related quotes is presented in Tables 4 and in Additional file 2: Table S1 presents supplementary quotes related to participants’ perceptions of returning genomic sequencing SFs about specific diseases and a VUS.

**Table 3:** Quotes related to participants’ perceptions of returning genomic sequencing SFs about specific diseases and a VUS

Disease/variant	Selected quotes
Hypercholester-olemia	Quote 5: “*We can adjust our diet according to that data. I have had high cholesterol since I was 18, and so, I adjusted my life with medication, training, healthy nutrition due to the diagnosis and the result I had. It was very beneficial to know it, and to make an adjustment, and avoid any coronary disease*”*.* Montreal male cancer survivor
	Quote 6: “*Me, I would rather not be worried about small things like that,…one day, I could be told “look, your lab results ( for cholesterolemia) are not good, I’m going to prescribe you a pill for it”…because I would already have cancer and all that…” Woman from the Quebec City general population*
Alzheimer’s disease	Quote 7: “…*but my first answer was ‘probably not’ (to receive a result showing that I’m carrying a genetic variant predisposing to Alzheimer's disease) because I would fear that it would poison my life for nothing. Because there is no treatment*.*”* Woman from the general Quebec City population
Wilson’s disease Cystic fibrosis VUS	Quote 8: “*On the other hand, for having experienced it, it may cause a problem with insurability. That is why I’m hesitant; I experienced it, I have children, and there are also cases of breast cancer in my family. There have already been some refusals of insurance coverage. My sons are young adults, but I would have a small hesitation. I agree with the whole issue of prevention, to contribute to research advancement, but I still have a small hesitation”*. Female cancer survivor from Quebec City Quote 9: “*Me, you see, to know that I was carrying a gene (pathogenic variant), before having children, I would have the man who was going to fertilize me tested, and we would decide not to have children”*. Female cancer survivor from Quebec City Quote 10: *“Since science is always in constant progression, we don’t know for now, but in five years from now, it may mean something. Maybe, we’re missing something crucial”.* Female cancer survivor from Quebec Quote 11*: “There is an anomaly, but they don’t know if it is serious. Why should I be informed about something that currently doesn’t cause any health problems? So, if I eventually get sick and it’s odd, well then, they can carry further research, but for now, I do not want to know about it. I tell myself it doesn’t give me anything to bother with it if they themselves don’t know what it is. An anomaly but almost everyone has one for sure, and we are doing pretty well, so I do not want to know about it.”* Woman from the general Quebec City population

**Table 4:** Participants’ motivations to receive or not to receive genomic sequencing SFs according to the disease or the type of SFs

Disease/ type of SFs	Reasons or motivations to receive SFs	Reasons or motivations to refuse the disclosure of SFs
Hypercholes-terolemia	-To make informed decisions to protect or improve their health through preventive and therapeutic measures -To be able to raise awareness among their families -Availability of treatments	-Not to aggravate cancer-related stress -Not a priority in the context of progressive cancer -Preference to receive such information after recovering from their cancer
Alzheimer’s disease	-To know whether there is a familial genetic predisposition to the disease -To be able to warn family members, ensure their support and understanding if, one day, they start having symptoms suggestive of Alzheimer’s disease -To get medical attention and supportive treatment earlier in the course of the disease, which hopefully could improve their survival -To be able to plan their career, live differently, enjoy life, leave memories, mentally prepare themselves and their relatives, make arrangements, and share wishes for their end of life (Table S1, Supplementary Quote 1) -To be able to take preventive or mitigating measures	-Absence of treatment or prevention -Lack of relevance for a cancer patient -Potential detrimental psychological impacts
Wilson’s disease	-To make informed decisions regarding reproduction -To help fast-track their children’s care in the case of an unexplained or hard-to-explain illness - To help orientate prospective familial genetic investigations and preventive or therapeutic measures in children (Table S1, Supplementary Quotes 2 & 3) - To be able to raise awareness and discuss the predisposition to Wilson’s disease with their families and healthcare professionals	-Fear that their children do not obtain adequate insurance coverage
Cystic fibrosis	- To be in full knowledge and ready psychologically - To be able to protect or warn their children - To be able to link children’s eventual symptoms to the disease - To make informed decisions about reproduction (Table S1, Supplementary Quote 4) - To inquire whether their children would be able to get insurance coverage	- Absence of treatment - Not to overwhelm as already affected by cancer - To keep living without having to worry about a predisposition to another disease - Not willing to be questioning themselves about whether to have children given that deciding not to have children would not be compatible with natural selection
VUS	- To benefit from related research and improve their survival in case they develop a related medical condition - To participate in research related to such variant - Potential starting point for familial genetic investigations and development of new treatment - Potential necessity for a specific follow-up for carriers of such variant - Preference for living while being aware of such variant even though more stressful - Less stressful, and easier to live while being aware of such variant - To be able to better understand the development of a disease related to such variant - To avoid missing on a finding that might become clinically relevant in the future - To contribute to knowledge advances and help the next generations (Table S1, Supplementary Quote 5)	-Too stressful and concerning to live with -The very nature of the VUS (absence of known health implications and therapeutic or preventive strategies) makes the communication of its existence irrelevant - Preference for that information to be documented in the medical chart and to be contacted once new data are available

In general, participants who were inclined to receive SFs wanted to make informed decisions regarding their health and, their families’ life in general, career, and reproductive options. They also wanted to raise awareness among their relatives and ensure family support in the event of disease. Participants who did not wish the return of SFs mentioned the potential negative psychological impacts, the absence of treatment for some diseases, and the difficulties in obtaining adequate health or life insurance coverage.

#### Hypercholesterolemia

The majority of participants agreed to receive SFs regarding hypercholesterolemia. The main reason was to obtain as much information as possible to decide whether to protect or improve their health through preventive and therapeutic measures (Quote 5). Those who were hesitant considered that a genetic predisposition to hypercholesterolemia was not a priority in the context of progressive cancer, and they would not want to have the additional stress (Quote 6).

#### Alzheimer’s disease

Most participants wanted to receive SFs revealing a genetic predisposition to Alzheimer's disease. This decision was motivated by the need to know whether there is familial genetic predisposition to the disease and the ability to warn potentially at-risk family members to ensure their support and understanding. They assumed that knowing their predisposition to this disease might prompt them to take preventive measures, get medical attention, and supportive treatment earlier in the course of the disease. On the other hand, other participants were less inclined to receive SFs related to Alzheimer's disease to avoid having it affect their life negatively (Quote 7).

#### Wilson’s disease

Most participants also wanted to know whether they were carrying a variant predisposing to Wilson’s disease. Such information would help them make informed decisions regarding reproduction. They believed that this information would help fast-track their children’s care in the case of an unexplained or hard to explain illness. One participant shared her concerns about the risks of not obtaining proper insurance coverage for her children due to a potential predisposition to Wilson’s disease. She reported having herself been denied insurance coverage due to her family history of breast cancer (Quote 8).

#### Cystic fibrosis

Participants generally agreed to receive SFs concerning cystic fibrosis. They wanted to be fully aware of the facts, be psychologically prepared, and make informed decisions regarding reproduction (Quote 9). Such information would also lead them to inquire about whether their children would get insurance coverage. Participants who did not want or were hesitant to know about a cystic fibrosis variant were motivated by the absence of treatment. They did not want to feel overwhelmed as they would already be affected by cancer. They would prefer to keep on living without worrying about being predisposed to another disease.

#### VUS

Opinions were particularly divided on the return of a VUS. Participants in favor of receiving such a result believed it could serve as a starting point for familial genetic investigations. Some participants said they preferred to live their lives aware of such a variant even though more stressful. Also, not being informed and tagged for a VUS might mean missing a finding that might become relevant in the future (Quote 10). However, those not in favor of receiving such information found it too stressful and concerning to live with. The very nature of a VUS (absence of known health consequences or potential therapeutic and preventive strategies) also makes the communication of its existence irrelevant to those participants (Quote 11).

The quotes related to the following subsections are shown in Table 5.

**Table 5:** Quotes related to participants' perceptions about the modalities of disclosing genomic sequencing SFs

Subtheme	Selected quotes
When to disclose genomic sequencing SFs?	Quote 12: *“The woman (in the case example) is in oncology, and she’s undergoing tests for something, and they discover another thing. I don’t know; there is also the moment. I don’t know if it’s the right time to tell her while she’s already overburdened with what she already has”* Woman from the general Quebec City population Quote 13: *“If the patient is in the terminal phase, it’s useless to overburden him with another disease…the patient is dying. He’s probably not even going to develop the disease depending on the stage of his cancer.”* Male from the general Montreal population Quote 14: “*I think that a physician faced with someone who is extremely anxious; I think that he should use his judgment. If the person were to get sick with anxiety at the thought of not knowing what they have or if it is going to kill them or not, I don’t think that does them any favors.* Woman from the general Quebec City population
Who should disclose genomic sequencing SFs?	Quote 15: “*Maybe the physician who ordered the test, you expect that he gives you the results that he requested, but maybe he could come a bit more prepared, especially in cases where they may be a little bit less like psychologists, at least, give you pamphlets or information or tell you “call this place or go to see this person, and they will be able to help you out if you have questions about them (SFs)*’…*”* Woman from the general Quebec City population Quote 16: “*In an ideal world, let’s say Alzheimer’s disease, the carrier gene, the oncologist could, even before meeting his patient, consult a specialist regarding this disease, and the Alzheimer’s specialist could take the time to explain to the patient, well, if you develop it (Alzheimer’s disease), (here are) the symptoms (that you could present). Same thing for Wilson’s disease. The oncologist could call a specialist in that disease.”* Woman from the general Quebec City population Quote 17: “*Let’s say the oncologist announces that I have a genetic abnormality predisposing to Alzheimer's disease, he won’t know how to answer all my questions. So, then, I’m going to see an Alzheimer's disease specialist. I tell myself, couldn’t the oncologist just transfer the information to the Alzheimer’s disease specialist, and I could meet him first instead of…Often; actually, we go down from one floor to another, and we walk around*.*”* Woman from the general Quebec City population Quote 18: “*Nothing prevents my family physician from getting my results and telling me. I meet him every year; I keep in touch with him.”* Montreal female cancer survivor

Who should disclose genomic sequencing SFs? (continued) How should genomic sequencing SFs be disclosed?	Quote 19: “*Of course, I would maybe like to meet him (a genetic counselor) to understand more, it’s that person’s world. He’s used to playing with the genes, and all that stuff, so, I would like that…I know there’s nothing serious, but I would like to understand and know the risks, and to learn more about it (secondary findings).”* Woman from the general Quebec City population Quote 20: “…*it should be the person who makes you undergo the tests. In my case, it was ordered by the surgeon, but it’s the geneticist who gave me the results. It must be him who transmits them…the person who makes you undergo the tests is in the best position (to transmit the results).”* Montreal female cancer survivor Quote 21: “Me, *I think that it would take a specialist who can communicate (several yes’s in the room), and who has time. No matter if it’s a physician or a nurse because if we look at the health care sector, there are nurses who are doing an outstanding job and who will take the time. They have more, I think, the opportunity to do so, but they are going to take more time with the patient than the physician. The physician has five minutes to tell you, and it’s ‘thank you, have a good day, see you next time’.”* Woman from the general Quebec City population Quote 22: “*But if it is something more serious like Alzheimer’s, Wilson’s disease or cystic fibrosis, whether it is a physician, a nurse or someone else, I would mostly want to have statistics or complementary information. What are the odds that you pass it on to your children? If it’s Alzheimer’s disease for you, for instance, what are the things that you can do to try to improve the situation? Take memory tests or play memory games or things like that. That’s that complementary information that I would like to have. I think that this should be done in person in this case”.* Male from the general population in Montreal Quote 23: “*Maybe it allowed me to keep control of my emotions or if I have a blackout, the other (the person accompanying me) can keep the information for me, and we can talk about it afterward”*. Montreal female cancer survivor Quote 24: “*Me, I go about it alone. After that, I take everything in, and after, I share what I wish to share*.*”* Montreal female cancer survivor Quote 25: “*For my partner, me, I would tell her more personally because I know her better, I know how to prepare her. In a fortuitous event, it’s more stressful to see her reaction than other things. Thus, I would prepare her calmly. I’m going to tell her, but I’m going to tell her more calmly. Otherwise, it will degenerate, I know that.”* Male from the general Quebec City population Quote 26: “*Me, one of the disadvantages that I would see…I think about the whole process (of my disease): the (cancer) diagnosis announcement, the treatments and all that…I didn’t want to have to deal with someone else’s sadness because I’m the strong one in the family…You all, deal with your sadness, me, I’m going to deal with mine*.*”* Montreal female cancer survivor Quote 27: “*I don’t know, I wouldn’t go that far because I already received MRI or scan results by phone, and that didn’t cause me any problem. I think if I have a risk of hypercholesterolemia, and they announce over the phone like that, it wouldn’t affect me at all*.*”* Montreal male cancer survivor

Themes	Selected quotes
Informing family members	Quote 28: “*I think that it’s the patient that must decide whether his family must know or not. There are some things that I wouldn’t tell my children, but if it implies that my children may have it later like Alzheimer’s, let’s say, I would tell them that I carry a gene (pathogenic variant), and maybe that you (her children) carry it also.”* Woman from the general population in Montreal Quote 29: “*The only thing I know is that with cystic fibrosis, and Wilson’s disease, I would say to the children, ‘check if you are not carriers also because if you have a partner who is a carrier, you have a one-in-four odds or two to have a child affected by a disease. It’s more for prevention.*” Woman from the general Quebec City population Quote 30: “*I am happy to be informed, but informing others is what scares me. I don't know their reactions. Informing other people makes me uncomfortable. Because we don't all have the same reactions. Some people panic.*” Quebec City male cancer survivor
Support needs	Quote 31: “*On the other hand, insurance companies should never know about it (genetic predisposition). It should be forbidden by law, and completely forbidden to discriminate based on genetic criteria …Based on observable criteria like do you smoke or not, things like that. Your gender, the geographic area where you live. All that is already proven, it’s already known, so why not just stop there and make ‘systematic’ laws…deny the insurance companies the right to ask for it (genetic information) and the option to set prices based on genetic information*.” Male from the general Montreal population Quote 32: “*Getting back to the law, should we also apply it to our employers? There are many employers who make you pass blood tests when you are first hired by a company…, but they can look for complementary information. They can tell themselves, ‘I’m not hiring him; he’s predisposed to this and that’.*” Male from the general Montreal population Quote 33: “*Maybe a support group, a phone number where you can call to have more information, a website…Something concrete that the person can go see, get informed, that the physician who conveys the result (genomic SFs) gives him all this information so that he’s able to leave from there (the office), and still feel a little, in control. I’m going to do that, I’m going to do that, and I’m going to do that.*” Montreal female cancer survivor

### When to disclose SFs?

Some participants expressed the need to be informed about SFs at the same time as the main results of the genomic sequencing are given. For cancer patients, the information received could allow for better cancer treatment planning. Other participants from the public preferred to deal with cancer first, and SFs disclosed after patient recovery. Several participants also believed that the attending physician should evaluate the patient’s profile and decide whether their age, psychological, emotional, and physical status or disease stage is compatible with genomic SFs communication (Quotes 12–14).

### Who should disclose SFs?

Participants thought that the prescribing physician, in this study, the oncologist, should communicate genomic SFs to the patients. As oncologists monitor cancer patients during their treatment, participants thought they should refer patients to family physicians or specialists (Quotes 15–17). Family physicians were perceived as capable of communicating SFs, particularly for hypercholesterolemia, as they know the patients well, and findings have no impact on the patient’s cancer management (Quote 18). According to some participants, family physicians could refer patients to a specialist or be accompanied by one when communicating the results. Nurses and psychologists were considered capable of discussing SFs with patients and supporting them after receiving such findings from physicians. Social workers were also regarded as capable of filling in for physicians. Some participants showed interest in consulting genetic counselors, as they were perceived as well-equipped to answer questions regarding disease risks (Quote 19). Several cancer participants shared their own genetic testing experience and explained that the geneticist that orders the DNA sampling and announces the results (Quote 20). They also thought that these results could be transmitted to the oncologist after being communicated at the genetics clinics. In general, participants thought it essential for healthcare professionals that inform patients about genomic SFs to be knowledgeable about genetic diseases, be a good communicator, show empathy, and have time to discuss (Quote 21).

### How should SFs be disclosed?

For a vast majority of participants, the best way to deliver SFs is during a face-to-face visit with a healthcare professional. Such a visit would allow patients to ask questions and obtain support and information on prevention and disease risks (Quote 22). Some participants wished to be accompanied by a friend or family member during their encounter with healthcare professionals. Such a companion might help the tested individual control his emotions and better understand the result implications (Quote 23). Other participants did not want to be accompanied as they viewed such visits as personal. Besides, they did not want to deal with family members' emotions and sadness (Quote 24–26). Several participants revealed that they did not mind receiving hypercholesterolemia-related SFs by phone, letter, email, or text message (Quote 27). Treatment availability, the relative ease in managing hypercholesterolemia, and its benign nature appeared to be the reasons behind this choice.

### Informing family members

Participants were divided about sharing genomic SFs with their relatives. Those who were in favor felt that the tested individuals should be the ones to make the decision about communicating their results to their relatives. In cases where there might be a risk to a tested individual’s offspring, these participants wished to inform their partner/spouse and their children (Quote 28). Once aware of the risks, the children and other family members could take preventive actions (Quote 29). These participants believed that physicians should strongly advise that tested individuals inform their relatives, particularly when the results involve disease risks to descendants. Several participants agreed that physicians could inform relatives but only after obtaining patients’ consent. A pre-written letter inviting family members to genetic counseling and testing was suggested to initiate discussions with relatives about genomic SFs. However, other participants believed that relatives should not be made aware of genomic SFs, especially if they are known to panic easily. One participant said that he felt scared and uncomfortable to share his results with his relatives due to their tendency to panic (Quote 30). Additionally, these participants thought that family members should be asked first whether they want to be informed about such findings.

### Participants’ support needs

Participants asked that patients be warned about the possibility of genomic SFs before undergoing the test. They wanted guidelines to be elaborated to harmonize patients’ management and genomic SFs. They were also in favor of laws or policies that would limit access to such findings to prevent insurance and employment discrimination (Quotes 31 & 32). Moreover, participants were interested in receiving information or documentation (pamphlet, booklet) on disease risks and prevention options. They also wished to have access to support groups, be guided towards psychosocial support resources, a dedicated website, or specialist physicians after the return of SFs or when the related disease occurs (Quote 33). They recognized that they might also need healthcare professionals' support to inform family members, like organizing a family meeting.

## Discussion

In this study, we sought to explore the perspectives of cancer patients and healthy members of the public about disclosing genomic SFs and the modalities of such disclosure. We found that most participants manifested a desire to receive SFs from genome sequencing for medical conditions that are amenable to treatment or not. There was an agreement among all participants that patients have the right to autonomously decide on the return or not of such findings, the type of SFs they want returned, and the extent to which they want to share them with other persons, including family members. Participants’ motivations to receive or not genomic SFs were both medical and personal and varied according to the disease’s treatability and severity. Regarding disclosure modalities, they considered that SFs be returned in a face-to-face visit by the prescribing clinician who should know about genetic diseases and have good communication skills. In the case of ongoing disease, they felt that SFs could be conveyed at the same time as primary results are divulged or after patient recovery. Finally, participants wished to see laws, policies or practice guidelines developed to protect confidentiality and prevent genetic discrimination.

To the best of our knowledge, this is one of the first studies to comprehensively explored (cancer) patients’ and lay people’s preferences about the modalities (the moment, the manner, the type of provider) of such disclosure. In that regard, our manuscript provides a unique contribution to this line of research and extends the findings of previous studies. We found little or no differences in viewpoints between cancer patients and participants from the public concerning the topics covered. This is consistent with previous studies where patients and lay individuals expressed the will to enjoy their right to autonomous decision-making and showed no disposition to rely on providers for the return of genomic sequencing SFs [18, 27–30]. In Brothers et al., patients wanted secondary variants to be analyzed and disclosed according to their preferences [31]. Patients’ freedom of choice is acknowledged in the ACMG 2016 policy statement. Professionals of this organization make the disclosure of variants not related to the primary indication of genomic sequencing conditional upon the patient’s consent the analysis [10]. Also, in some previous studies, genetics specialists seemed inclined to give tested individuals the choice to know or not and to abide by their decision regarding the disclosure of SFs [32–34].

In previous studies, patients, parents of children affected by a genetic condition, adolescents, research participants, and individuals from the general population were generally favorable to disclosing genomic SFs [20, 28, 35–44]. The justification behind this preference were similar to those formulated by the participants in the present study. They included the option to take preventive or therapeutic actions, make an informed decision regarding different aspects of their life (career, reproduction, familial support, leisure), and contribute to research advancement [27, 28, 42, 45]. As desired by participants in the present study, parents of affected children and lay individuals in other studies wanted to learn about SFs during a consultation with a healthcare professional knowledgeable of the discovered pathogenic variants, the disease it predisposes to, and its genetic components [28, 38]. This professional should also use clear and comprehensible language. Parents of children affected with rare diseases in Kleiderman et al. also wanted result recipients to benefit from psychosocial support when needed [38]. Consistent with the present study's findings, not all participants in previous studies wanted to receive genomic SFs [28, 39, 46, 47]. The potential negative psychosocial impacts, and the lack of clinical utility were also part of the rationale behind such a stance [27, 28, 39, 42, 46, 47]. Furthermore, concerns regarding the confidentiality of the results and genetic discrimination were also expressed in other studies by non-healthcare professionals [27, 28, 43].

Personal or familial experiences with a disease may influence tested individuals’ propensity to receive SFs in different ways. Indeed, participants already suffering from hypercholesterolemia in this study particularly welcomed the idea of returning SFs related to such disease. Moreover, a cancer patient wanted to have SFs returned to ensure that her body was running well despite several comorbidities. However, a participant from the general population, seemingly proud of having survived several serious diseases, reported that he did not want to hear anything about having a predisposition to an additional disease. In the same vein, most hereditary cancer patients in Houdayer et al. (2019) were unfavorable to the search for SFs. They did not want to live through another announcement as traumatic as their diagnosis [30]. However, in the same study, parents of children affected by an undiagnosed disease showed high interest in receiving SFs that might have helped to elucidate the origin of their children’s disease [30].

Some participants in the present study suggested that SFs be disclosed by the family physician and the disease specialist. For their part, genetics, legal and bioethical experts in Gourna et al. (2016) considered that SFs should be disclosed by a multidisciplinary team comprised of several specialists capable of offering comprehensive management to the patient [48]. In the absence of such a team, as some participants in the present study, these experts perceived genetic counselors as well prepared to disclose and discuss genomics SFs with patients. Participants in the present study also wished that clinicians ordering genome sequencing discuss potential SFs with them before proceeding. However, it should be noted that insufficient knowledge in genetics has been reported in non-genetics health professionals in several studies [49, 50]. Non-genetic physicians expressed concerns regarding such lack of knowledge and the fast-growing body of knowledge on genomic medicine [51]. They were also found to be unprepared to order genomic tests, interpret and convey the results, and integrate genomic information in their clinical practice routine [52, 53]. Several education initiatives intended for such professionals have been put in place, but their impacts on genetics/genomics clinical mainstreaming have not been examined [54]. This suggests that scaling up the return of genomic SFs might be challenging. Furthermore, healthcare professionals involved in disclosing genomic sequencing results have identified additional challenges when announcing genomic SFs. They reported having to deal with patients’ high expectations regarding the results and having to return multiple unexpected and uncertain results as well as discussing rare diagnoses [55]. In Appelbaum et al. (2014), patients thought that, while obtaining their consent to the test, clinicians should also discuss disease prevention options, eventual errors in the interpretation of variants, potential psychological impacts, and the evolution in variant significance with scientific advances [56].

This study presents some strengths and limitations. The double coding of discussion transcripts by two different researchers and consensus findings increases the study results' validity. This study allowed for gathering the perception of a relatively large sample about the disclosure of SFs in the hypothetical context of progressive and drug-resistant cancer. The qualitative approach allowed participants to justify their points of view. The focus groups were conducted by a neutral moderator, which allowed participants to freely express their opinions. However, as a non-medical professional, the moderator could only provide participants with a limited amount of information concerning symptoms, clinical impacts, and prognosis of diseases not well known to participants among those proposed in the vignettes. Such information could have better informed the participants’ viewpoints and might have impacted their opinions since the perceived severity of a medical condition may play a role in potential recipients’ decision to learn or not about related SFs [47, 57]. Study participants provided their viewpoints on a hypothetical drug-resistant cancer with five vignettes on potential SFs. The possibility that their stance and attitudes differ when faced with a similar situation in a real clinical setting cannot be ruled out. Since participation in the study was voluntary, it is not known to what extent the views of study participants are generalizable. Nevertheless, this is a qualitative study, and the generalisability of the findings was not the chief objective here. Finally, to make it easier for participants we presented the vignette related to the VUS in a general term, although we acknowledge that a VUS can have very different implications depending on what type of gene [58].

## Conclusion and implications for practice and research

In general, we found that both cancer patients and lay individuals were favorable to face-to-face disclosure of SFs. However, this should be seen as aspirational, given that several participants revealed that they did not mind receiving hypercholesterolemia-related SFs for example by phone, letter, email, or text message. Likewise, we do not believe that systematic face-to-face disclosure is a realistic way to scale up the return of SFs. The main reason for that is the shortage of genetic counsellors [59, 60] and the lack of preparedness of other health care professionals, particularly primary care physicians [61]. Patients’ autonomy, the right to decide on the return or not of SFs and the type of SFs they wish to receive were considered essential. Adverse psychological impacts represented the main drawback or dissuasive factor to such disclosure. The willingness to be informed about SFs appears to depend on participants’ values, disease experiences and perceptions, priorities in life, and self-perceived ability to endure negative psychological effects. Modalities for prescribing genomic sequencing in clinics, providing genetic counseling, and disclosing SFs should accommodate patients’ preferences and needs. Professional societies and lawmakers could draw from our results and those from others to elaborate nuanced recommendations and regulations that focus on standardizing clinicians’ approach and preserving patients’ and families’ autonomy and wellness. Considering SFs while referring patients to genetics clinics for counseling, communicating and managing such findings would undoubtedly increase the healthcare professionals’ workload, the time spent with patients, and healthcare system expenditures [62]. Thus, adjustment might be needed in healthcare systems funding. Genetic counseling and laboratory genomics reports could also be adapted to patients’ educational level and medical literacy [29, 48, 63].

## Supplementary Information

**Additional file 1**. This document presents the discussion guide.

**Additional file 2**. Table S1 shows supplementary quotes related to participants.

## Data Availability

Group discussions transcripts generated during the current study are not publicly available as per Ethics requirement that we maintain confidentiality of participants. We provided the interview guide in the Supplementary Document 2.
