# Supplementary material for: Whether, when, how, and how much? General public’s and cancer patients’ views about the disclosure of genomic secondary findings
Source: BMC Med Genomics. 2021 Jun 26;14:167. doi: 10.1186/s12920-021-01016-8 (PMC8236159; doi:10.1186/s12920-021-01016-8)
Supplement: Supplementary file 1 — Additional file 1. This document presents the discussion guide. [file 12920_2021_1016_MOESM1_ESM.docx]

**Table S1. Supplementary quotes related to participants’ perceptions of returning genomic sequencing SFs about specific diseases and a VUS**

|  | Selected quotes |
| --- | --- |
| Alzheimer’ Disease | Supplementary Quote 1: “*I would prepare my exit, I would make it so that I leave memories of me so that my children and people around me remember me in a more concrete way than if I leave like that, and I don’t remember nothing.”* Woman from the general Quebec City population |
| Wilson’s Disease | Supplementary Quote 2: “*Most of the time, when we want to know it (a genetic predisposition to a disease), it’s more for our children and grand-children for prevention. Often, it’s mostly for that*.*”* Woman from the general Quebec City population |
|  | Supplementary Quote 3: “*To protect the children…You always want to protect your children. If you know it (a genetic predisposition to a disease), you can ensure that, if there is something, an effective treatment, the children benefit from it”*. Female cancer survivor from Quebec City |
| Cystic fibrosis | Supplementary Quote 4: “*We are carriers of a gene (pathogenic variant), and my brother met someone who is also a carrier, and we have children diagnosed with cystic fibrosis. So, I’m a bit biased. It is a shock in the family for sure, but we were happy to know that the gene existed. For one, to protect the children because it’s a deadly disease, it is not easy to treat and for the future also. We have children, and children meet other people (with whom they may have children)”*. Female cancer survivor from Quebec City |
| VUS | Supplementary Quote 5: *When we stop and think about it, for the same reasons, no matter how small they are, I tell myself I'm going to move something forward. To me, that won't change much. It will change something for my children or in the future. I always come back to that. They are the ones who will benefit from it afterward. Now, we do with what we know, but later, how far will our knowledge go? We do not know.* Female cancer survivor from Montreal |
