# Supplementary material for: Whether, when, how, and how much? General public’s and cancer patients’ views about the disclosure of genomic secondary findings
Source: BMC Med Genomics. 2021 Jun 26;14:167. doi: 10.1186/s12920-021-01016-8 (PMC8236159; doi:10.1186/s12920-021-01016-8)
Supplement: Supplementary file 2 — Additional file 2. Table S1 shows supplementary quotes related to participants. [file 12920_2021_1016_MOESM2_ESM.pdf]

Preferences regarding the disclosure of secondary findings from clinical genomics.

DISCUSSION GUIDE

Maximum expected duration: 2 hours

<u>IMPORTANT</u>

In order to cover all questions in the allotted time, it is important to respect the time assigned for each section. Strict adherence to the sections is essential to ensure consistency and quality in data processing.

Version : January 18, 2018

PROCESS

1. WELCOME

- Participants settle in
- Time to complete the consent form and the socio-demographic questionnaire

2. INTRODUCTION TO THE DISCUSSION (15min)

- Moderator introduction
- Roundtable
- Thank the participants
- Purpose of the group discussion
- Introducing the terms genomics and secondary findings
- Invitation to speak freely
- Group discussion rules
- Confidentiality of the discussion
- Role of the moderator and research team

3. QUESTIONNAIRE (1h30)

- Scenarios : presentation of fictitious cases
- Opinions and attitudes
- Disclosure method preferences
- Perceptions of psychosocial impacts
- Support and guidance for decision-making on whether or not to know the results of secondary findings
- Time to fill-out the questionnaire on preferences

4. SUGGESTIONS AND OTHER QUESTIONS (10 min)

5. CONCLUSION (5 min)

1. WELCOMING PARTICIPANTS

Participants will be welcomed at the premises of the SOM firm in Montreal and Quebec. These premises are equipped with all the material required for conducting group discussions. Participants will be invited to sit in the discussion room and complete a brief socio-demographic questionnaire. A tent card with the first name will be placed in front of each participant to facilitate discussions. A sheet of paper will also be given out so that participants can write down ideas while waiting their turn to speak.

2. INTRODUCTION TO THE DISCUSSION

Moderator introduction *(name, first name, company for which she works, her experience, etc.)*

Roundtable to allow participants to introduce themselves

First of all, thank you for agreeing to participate in this research project conducted by researchers from the CHU de Québec and CHU de Montréal research centers and McGill University. This project, entitled "Societal preferences concerning the disclosure of incidental discoveries resulting from clinical genomics", receives financial support from the Social Sciences and Humanities Research Council of Canada (SSHRC) and Genome Canada and has received approval from the Research Ethics Committees of the CHU de Québec and the CHU de Montréal.

Before we start the discussion, I would like to know if you have ever heard of GENOMICS (Wait for the reaction before moving on with the following).

In fact, you probably know more than you think. For example, you have certainly heard about DNA and genes in the media or from your entourage. Likewise, you are surely aware that we have all inherited several traits from our parents, grandparents and ancestors (eye color and hair color, height for example). These traits are dictated, at least in part, by our genes which are responsible for the proper functioning of our organism. For this reason, an abnormality in the genes can, but not always, cause a genetic disease. Globally, this is what we call genomics, which is the study of how our genes work.

<u>**Make sure everyone is doing fine before continuing?**</u>

For this reason, doctors are increasingly using genetic information to diagnose or prevent certain diseases or to guide the treatment of patients. Today, thanks to advances in research on the human body and computer science, it is possible to read the information of THOUSANDS of genes in record time at a relatively affordable cost. Although this new technique offers potential benefits for patients, its use could also pose problems.

In fact, by analyzing multiple genes at the same time, doctors can “accidentally” discover information they weren't initially looking for. This information may relate to our origins, but it can also tell us that we could suffer from serious illness in the future. The important question that is currently being asked in Quebec, Canada and elsewhere in the world is the following: Should physicians disclose this unexpected information to their patients?

For the moment, opinions are very divided among professionals and health authorities. However, the voices of the public, citizens and patients have yet to be heard. This is why researchers from the CHU

de Québec-Université Laval Research Center and the CHUM Research Center set up this research in order to collect your opinions and thoughts on this issue.

As mentioned when you were approached, the discussion will last a maximum of two hours.

There are no right or wrong answers, only different points of view. There is also no need to seek consensus. Everyone should feel comfortable giving his/her opinion.

To facilitate the discussion, please speak loudly and only one person at a time. For your information, a few research team members are located in another room where they can observe and hear the group discussion. In addition, we will be recording the discussion because we do not want to lose any of the opinions expressed. We will only use your first names for discussion purposes, but these will be replaced by fictitious names when the researchers analyze their data and produce their results. Be assured that your confidentiality will be respected and preserved.

Today's discussion will take place in three parts. The first part is a scenario during which I will present a fictitious but realistic case to illustrate what we will discuss. In the second part, I will ask your thoughts on the issue of disclosing secondary findings obtained in the context of genetic analysis. In the third part, I will invite you to ask any other questions or to share with us other aspects that you would like to address.

3. QUESTIONNAIRE

**Scenario: presentation of a fictitious case**

I would now like to present a scenario to you through a fictitious case, but that could happen in real life. This scenario will be useful for you to answer the questions in the next section. **(*Show text on screen* :)**

Mrs. (or Mr. depending on the group) X is 40 years old. She was diagnosed with colorectal cancer (cancer of the colon and rectum). Unfortunately, Mrs. X developed treatment resistance. In order to understand the reasons for this resistance and possibly reorient the treatment, her oncologist requires an in-depth analysis of her genes. The results of the comprehensive gene analysis do not show any abnormalities in the genes associated with colorectal cancer. However, these results indicate that :

VIGNETTE 1

Mrs. X has a genetic mutation that puts her at greater risk for having high cholesterol. There are currently effective treatments for high cholesterol.

VIGNETTE 2

Mrs. X has a genetic mutation that puts her at greater risk of developing Alzheimer's disease. Although there are medicines to reduce some symptoms, they do not prevent or slow the progression of this fatal disease.

VIGNETTE 3

Mrs. X has a genetic mutation for Wilson's disease (an inherited genetic disease that prevents the elimination of copper from the body). She carries the mutation for this disease but is not affected. However, her children could be affected by the disease if their father has the same mutation in this gene. There are effective treatments to cure this disease.

VIGNETTE 4

Mrs. X has a mutation in the gene that causes cystic fibrosis, a fatal disease that affects various organs (lungs, digestive tract) in children and young adults. Like Wilson's disease, she carries the mutation but does not have the disease. However, her children could be affected by the disease if their father has the same genetic defect. There is no cure.

VIGNETTE 5

Mrs. X has a genetic variant of unknown significance, that is to say, that this mutation can cause illness for her or her relatives, but the current state of research does not allow this to be known.

Semi-structured interview guide (focus group period)

We will now move on to the actual discussion. I am therefore going to ask you a number of questions which you can answer, keeping in mind the vignettes I have just presented to you.

Section 1: Opinions and Attitudes

a. In general, what do you think of the idea of giving Mrs. X the choice of knowing her results?
b. Based on the fictitious case presented above, for which situation (s) would you like to know (or not know) your results?
c. What are the factors or reasons that would motivate your decision to know or not to know about your secondary findings if you were faced with this situation?

* Note: If the participants do not provide a sufficiently satisfactory answer to these questions, it is suggested that the moderator reformulates the question making sure they are fully understood.

Section 2: Disclosure Method Preferences

(* Note to the moderator: It is important to let the participants think by themselves before offering possible answers).

a. How do you think the secondary findings should be disclosed to affected patients?

Proposed answers (if necessary)

- ***In person***
- ***By phone***
- ***By email***
- ***By letter***
- ***In a group***

b. Should we inform relatives or blood relatives? How?
c. Should the mode of disclosure depend on whether the disease risk is curable? Why?
d. Who do you think (which healthcare professional) should disclose the secondary findings to patients and their families?

Proposed answers (if necessary)

- ***The specialist doctor***
- ***The family doctor***
- ***A specialist nurse***
- ***A non-specialist nurse***
- ***A genetic counselor***
- ***Others***

e. In your opinion, should the healthcare professional who discloses the secondary findings be different depending on the nature (curable or not) of the disease risk? Why?

<u>Section 3 : Perceptions of Psychosocial Impacts</u>

a. What would be your concerns or worries about the secondary findings?
b. In your opinion, what would be the risks and benefits of knowing (or not) these secondary findings?

<u>Section 4: Support and guidelines regarding the disclosure of secondary findings</u>

a. Do you believe that the disclosure of secondary findings should be regulated or not by law or professional recommendations (guidelines)? I would ask you to clarify your answer.
b. What kind of information or support would you need to help you with your decision if you were faced with such a situation?
c. Do health professionals have a role to play? Which one?
d. Would you need to be accompanied by a loved one if you were in a situation where you were expected to have secondary findings?

Before concluding, we would like you to individually complete the short questionnaire about your preferences regarding whether the opportunity to disclose or not secondary findings in each of the situations we have just discussed.

4. SUGGESTIONS AND OTHER QUESTIONS

a. Do you have any questions that may not have been addressed in today's discussion?
b. Do you have any additional information that you would like to share that was not covered during the meeting?

5. CONCLUSION

- Thanks.
- Reminder on the confidentiality of discussions and data.
